# Effect of Pattern and Duration of Breastfeeding on the Consumption of Fruits and Vegetables among Preschool Children

**DOI:** 10.1371/journal.pone.0148357

**Published:** 2016-02-04

**Authors:** Betina Soldateli, Alvaro Vigo, Elsa Regina Justo Giugliani

**Affiliations:** Universidade Federal do Rio Grande do Sul, Hospital de Clínicas de Porto Alegre, Porto Alegre, Rio Grande do Sul, Brazil; Hamamatsu University School of Medicine, JAPAN

## Abstract

**Background:**

The duration and pattern of breastfeeding can influence the consumption of fruits and vegetables in later childhood.

**Objective:**

To investigate the association between pattern and duration of breastfeeding and consumption of fruits and vegetables in children aged between 4 and 7 years.

**Methods:**

We conducted a secondary analysis using data from a former randomized clinical trial with 323 adolescent mothers, their children, and maternal grandmothers, when they cohabited. Information on infant feeding was collected monthly during the first 6 months of life, every two months until the child was 1 year old over and when children were between 4 and 7 years old. The associations between duration of breastfeeding and exclusive breastfeeding and consumption of fruits and vegetables were tested by a logistic regression model.

**Results:**

Approximately 60% and 45% of children consumed fruits and vegetables, respectively, five or more times a week. Consumption of vegetables among 4-7-year-old children was higher in children who were breastfed for 12 months or longer (OR 2.7; 95%CI 1.49–4.93); however, exclusive breastfeeding duration did not have a significant association with consumption of vegetables (OR 1.5; 95%CI 0.70–3.04). There was no association between weekly consumption of fruits and duration of breastfeeding (OR 1.3; 95%CI 0.71–2.30) or exclusive breastfeeding (OR 0.7; 95%CI 0.34–1.44).

**Conclusions:**

Longer duration of breastfeeding was positively associated with consumption of vegetables in children aged 4–7 years; however, there was no association with consumption of fruits. Exclusive breastfeeding duration did not have influence on consumption of fruits or vegetables.

## Introduction

Consumption of fruits and vegetables are essential for a healthy and high-quality diet. These foods are related to the prevention of diseases such as childhood obesity [1]. The Brazilian Ministry of Health recommends that children older than 2 years old should eat fruits on a daily basis. In addition, vegetables should be included at least in two main meals [2]. Such recommendation also aims to reduce the consumption of energy-dense and nutrient-poor foods. However, national data show that this recommendation is not followed by most Brazilian children [3].

Eating habits are established during the first years of life and affect all stages of the life cycle [4–6]. A complex network of genetic and environmental factors participate in the formation of eating habits, which begins in the prenatal period through the foetus contact with the amniotic fluid [7]. After birth, early eating experiences become part of this network, such as breastfeeding, which, according to some authors, would have influence on food acceptance by children. Breastfed children are exposed to a variety of flavours that reflect their mother's diet, making them familiar with the family's eating habits [8]. Some studies have suggested that breastfeeding pattern and duration may influence the consumption of fruits and vegetables in preschool children [9–13]; other, however, have not shown this association [14].

Our study aims to increase knowledge about the role of breastfeeding in the dietary pattern later in childhood. With that purpose in mind, we intended to answer the following question: is there an association between the pattern and duration of breastfeeding and the frequency of consumption of fruits and vegetables in a sample of Brazilian preschool-aged children?

## Methods

In order to answer this question, we used data from a randomized clinical trial aimed at evaluating the effect of an intervention on the prevalence of exclusive breastfeeding in the first 6 months of life, as well as the prevalence of breastfeeding and healthy complementary feeding in the first year of life. This clinical trial was conducted, with 323 adolescent mothers, their children, and their mothers (maternal grandmothers) when they cohabited. Adolescent mothers were recruited between 2006 and 2008, in the maternity ward of the Hospital de Clínicas de Porto Alegre, a public teaching hospital, with approximately 3,000 births every year. The inclusion criteria were mothers younger than 20 years old, living in the city of Porto Alegre, who had given birth to healthy babies with birth weight was ≥ 2,500 g and who were being breastfed. The intervention consisted of six counselling sessions on breastfeeding and healthy complementary feeding. The first session was held in the maternity ward and the remaining sessions took place at the participants’ homes at 7, 30, 60, and 120 days of life. Infant feeding information was collected monthly for the first 6 months, every two months until the child turned 1 year old, and between 4 and 7 years of age. Such data were collected by telephone interview with the mothers or during home visits when a phone interview could not be carried out, except for the last assessment. When children were between 4 and 7 years old, their mothers were contacted and invited to attend the Centre of Clinical Research of the Hospital de Clínicas de Porto Alegre. They were supposed to take their children along. When mothers and their children could not attend the Centre, we arranged for a home visit. During these visits, the mothers answered a questionnaire about usual weekly food frequency (FFQ). The results and further details of this randomized clinical trial were reported elsewhere [15–17].

The group of vegetables included a wide variety of garden crops, except for potato and cassava, which were classified as tubers. Fruit juice was not included in the fruit group.

Children on exclusive breastfeeding were those receiving only breast milk without any other type of liquid or solid food, including water, tea, and juice. Children being breastfed were those receiving breast milk regardless of whether they received other foods or not. In spite of the recommendation of providing exclusive breastfeeding for 6 months, exclusive breastfeeding duration was categorized as ≥ 4 months and < 4 months, because the prevalence of women that follow the recommendation is very low. The duration of breastfeeding was categorized as ≥ 12 months and < 12 months. We decided to use this cut-off point because 12 months is the approximate median duration of breastfeeding in Brazil (11.2 months) [18].

Data were computer stored by means of a double data entry method and later validation using Excel^®^. Statistical analysis was performed using the Statistical Analysis System (SAS 9.4). Quantitative variables were described as mean and standard deviation or median and interquartile range. Categorical variables were expressed as absolute and relative frequencies. Means were compared using the Student t test or Mann-Whitney test, whereas proportions were compared using Pearson's chi-square test or Fisher's exact test.

Initially, we compared the characteristics of the participants who completed the study with the characteristics of those who were lost to follow-up. Next, the association between duration of breastfeeding and exclusive breastfeeding and weekly consumption of fruits and vegetables at the recommended frequency (five or more times a week) was tested using a logistic regression model. We first tested the unadjusted model (model 1), and then constructed two cumulative models. The model 2 considered the initial design of the study, i.e., the children's group (control or intervention); and model 3 included some variables that are considered having influence on children's diet, such as maternal education, number of siblings, child's gender and age, and cohabitation with maternal grandmother [19–21]. The effect measure we used was odds ratio (OR) in combination with 95% confidence interval.

This study was in compliance with the Standards of Health Research. Mothers or guardians were informed about the study and, after agreeing to participate, they signed a written consent form. When adolescents were younger than 18 years old, after agreeing to participate in the research, the informed consent was signed both by them and their guardians. This study was approved by the Scientific Committee and Research Ethics Committee of the HCPA (n° 120249). The clinical trial was registered at http://www.ClincalTrials.gov, identifier: NCT00910377.

## Results

Of the 323 mothers and children who started the study, 257 (80%) stayed in the study until the 6th month of children’s life, 219 (68%) remained up to 12 months, and 207 (64%) were still participating in the study in the last evaluation (4–7 years). Some participants were lost during the study because mothers/families could not be reached (n = 91), there was refusal to remain in the study (n = 23), separation of mother and infant (n = 1), and mother's death (n = 1).

The distribution of the participants' characteristics was balanced between those who completed the study and the participants who were lost to follow-up. There was no statistically significant difference (p<0.05) in terms of maternal characteristics (race, age, income, educational level, number of prenatal visits, and number of children), children's characteristics (gender, birth weight, and mode of delivery), and mothers’ cohabitation with their own mothers and partners. The number of losses was similar among the participants allocated to the intervention group– 56% (n = 65)–and the control group 44% (n = 51) (p = 0.167).

Children's mean age in the last evaluation was 6.1 years (4.7–7.0). Participants' characteristics in the last evaluation were as follows: mean *per capita* family income = 0.6 minimum wage; maternal years of school ≥ 8 years = 45.4%; primiparity = 62.3%; mother with job = 54.1%; male child = 48.8%; cohabitation with husband/partner = 64.3%; cohabitation with maternal grandmother = 26.6%.

Approximately half of children (52.7%; n = 109) had been breastfed for at least 12 months and 21.3% (n = 42) had been exclusively breastfed for at least 4 months. Among children aged 4–7 years old, approximately 60% consumed fruits almost every day (5 or more times a week) and 45% consumed vegetables at the same frequency. Table 1 shows the weekly consumption of fruits and vegetables among children of that age group, according to the duration of breastfeeding and exclusive breastfeeding.

**Table 1:** Frequency of weekly consumption of fruits and vegetables among children between 4 and 7 years old according to the duration of BF and EBF.

Weekly consumption	Fruit consumption						Vegetable consumption					
	EBF			BF			EBF			BF		
	<4	≥4	P	<12	≥12	P	<4	≥4	P	<12	≥12	P
	n = 155	n = 42		n = 98	n = 109		n = 155	n = 42		n = 98	n = 109	
Almost every day	63.2%	50.0%	0,254	58.2%	61.5%	0,384	42.6%	50.0%	0,285	33.7%	55.0%	0,03
3 to 4 times a week	25.8%	38.1%		27.6%	30.3%		20.6%	26.2%		22.4%	21.1%	
≤ 2 times a week	11.0%	11.9%		14.3%	8.3%		36.8%	23.8%		43.9%	23.9%	

BF = breastfeeding; EBF = exclusive breastfeeding; Almost every day = > 5 times a week.

The weekly consumption of vegetables was significantly higher among infants who were breastfed for 12 months or longer; the same was not true for the consumption of fruits (Table 2). The duration of exclusive breastfeeding was not associated with the consumption of vegetables or fruits (Table 3).

**Table 2:** Duration of breastfeeding and weekly consumption of fruits and vegetables at 4–7 years old.

		Weekly fruit consumption OR (95%CI)	Weekly vegetable consumption OR (95%CI)
Model	BF duration	≥ 5 times	≥ 5 times
Model 1	< 12 months	1	1
	≥ 12 months	1.2 (0.68–2.11)	2.6 (1.45–4.56)
Model 2	< 12 months	1	1
	≥ 12 months	1.2 (0.69–2.14)	2.6 (1.44–4.54)
Model 3	< 12 months	1	1
	≥ 12 months	1.3 (0.71–2.30)	2.7 (1.49–4.93)

Model 1: not adjusted; Model 2: adjusted for intervention; Model 3: adjusted for intervention, child's age and gender, mother's educational level, cohabitation with the maternal grandmother, and number of children.

**Table 3:** Duration of exclusive breastfeeding and weekly consumption of fruits and vegetables at 4–7 years old.

		Weekly fruit consumption OR (95%CI)	Weekly vegetable consumption OR (95%CI)
Model	EBF duration	≥ 5 times	≥ 5 times
Model 1	< 4 months	1	1
	≥ 4 months	0.6 (0.30–1.22)	1.4 (0.69–2.74)
Model 2	< 4 months	1	1
	≥ 4 months	0.6 (0.32–1.31)	1.3 (0.65–2.68)
Model 3	< 4 months	1	1
	≥ 4 months	0.7 (0.34–1.44)	1.5 (0.70–3.04)

Model 1: not adjusted; Model 2: adjusted for intervention; Model 3: adjusted for intervention, child's age and gender, mother's educational level, cohabitation with the maternal grandmother, and number of children.

## Discussion

The present study showed a positive association between duration of breastfeeding for 12 months or longer and greater consumption of vegetables among children aged 4–7 years old. However, we could not find any association between duration of breastfeeding and consumption of fruits, as well as between duration of exclusive breastfeeding and consumption of vegetables and fruits.

Other studies have also shown a positive association between duration of breastfeeding and consumption of vegetables in later childhood. Perrine et al. (2014) investigated the association between duration of breastfeeding and several markers of diet quality at 6 years of age in a sample of 1,355 U.S. children. These authors found that children who were breastfed for 12 months or longer were more likely to consume more vegetables than the median daily rate of vegetable consumption at 6 years of age (OR 1.65; 95%CI 1.14–2.40). However, unlike the present study, they found a positive association between duration of breastfeeding and fruit intake (OR 2.09; 95%CI 1.40–3.11), as well as between duration of exclusive breastfeeding ≥ 3 months and consumption of fruits and vegetables (OR 2.03; 95%CI 1.34–3.06 and OR 1.45; 95%CI 1.00–2.13) when compared with children who were breastfed for less than 3 months or never breastfed [11].

A recent analysis of four European cohorts of children aged 2 and 4 years found an association between breastfeeding for 6 months or longer and greater consumption of vegetables in the four cohorts (UK, France, Greece, and Portugal); however, in just two countries (UK and France), breastfeeding for 6 months or more was associated with greater consumption of fruits [9].

A possible explanation for the increased consumption of fruits and vegetables by children who are breastfed for longer could be that these children probably are more exposed to a greater variety of flavours early in life through breast milk [7]. Consequently, they might have greater acceptability of new foods during the introduction of complementary feeding [8] which facilitate better eating habits later. However, we cannot rule out the possibility that women who follow the recommendation to breastfeed for a longer period also try to follow the other recommendations in terms of offering a healthier diet to their children. These women themselves may have healthier lifestyles and eating habits; therefore, they provide their children with the healthy foods that they consume. The study of Khalessi and Reich [22] reinforces this assumption. They found that women who are committed to breastfeeding may also be more committed to making healthier feeding choices. Future research should explore if there are some characteristics in breastfeeding mothers that predisposes them to make healthier feeding choices for them and their children. Future studies should also examine if mothers with healthier eating habits tend to breastfeed longer.

Conversely, our findings did not confirm the association between longer duration of exclusive breastfeeding and higher consumption of vegetables later in life, which was found in other studies [10–12]. However, the method used to measure the consumption of food was different in each study: frequency of portions according to Canada's Food Guide (Burnier), grams per day (Moller), median daily intake (Perrine), and weekly frequency (our study). Such differences may have contributed to the discrepancy in the results.

Another point to consider is that the mother should initiate breastfeeding to be included in the study. This might be likely different from some of the other studies, contributing for differences in results. However, in Brazil more than 95% of women start breastfeeding, including adolescent mothers [23]. Therefore, we believe that our results would not be different if we had included the very few mothers that did not initiate breastfeeding.

Some limitations of the present study should be pointed out. Active search for the children included in our study did not ensure fewer losses. However, such loss rate is expected in studies requiring follow-up, especially when involving young adults living in the outskirts of large cities in developing countries. Nevertheless, this limitation was minimized by the fact that the characteristics of the lost participants were not different from those of the participants who completed the study.

The food frequency questionnaire did not include the option of daily consumption of the foods. As a result, we could only test the association between duration of breastfeeding and “almost every day consumption” of fruits and vegetables. Also, we only measured the weekly frequency of consumption of fruits and vegetables, instead of evaluating the amount of food consumed. We are aware of the fact that the frequency of food consumption does not reflect the actual intake of food consumed in an accurate manner. However, other authors [14] also found no association between duration of exclusive breastfeeding and consumption of fruits and vegetable in a sample of Brazilian pre-schoolers, despite using portions (grams per day) to measure food consumption. On the other hand, positive associations between duration of breastfeeding and consumption of fruits and vegetables were found in some studies when outcomes were measured more accurately [10–12].

Another important aspect that should be considered is the wide age range of children (4 to 7 years) in assessing the outcome. However, we believe that this aspect did not have a significant impact on the results because the children's age was included in the analysis model. In addition to the children's age, our model also considered some variables that were specific of the study design, such as cohabitation with the maternal grandmother and group to which the participants were allocated. The results showed that probably the intervention did not affect the results. Also, we already showed that the variables cohabitation with the grandmother and intervention had no effect on the quality of children's diet at 4–7 years old [17].

In conclusion, although our study has confirmed the association between longer duration of breastfeeding and higher consumption of vegetables in preschool age, our findings can not support the hypothesis that breastfeeding itself promotes better eating habits in future stages of life, since we did not confirm the association between longer duration of breastfeeding and higher fruits consumption or longer duration of exclusive breastfeeding and higher vegetable and fruit consumption. It seems that the experience with breastfeeding alone is not sufficient to enhance healthy food habits in older children, since infants must taste the food to learn to like it, and it depends on the healthy feeding offer, being encouraged and available by a family context.
